# New Insights in Molecular Mechanisms and Pathophysiology of Ischemia-Reperfusion Injury 2.0: An Updated Overview

**DOI:** 10.3390/ijms22010028

**Published:** 2020-12-22

**Authors:** Arnau Panisello-Roselló, Joan Roselló-Catafau, René Adam

**Affiliations:** 1 Ischemia-Reperfusion Unit, Experimental Pathology Department, Institut d’Investigacions Biomèdiques de Barcelona (IIBB)-IDIBAPS, Spanish Research Council (CSIC), 08036 Barcelona, Catalonia, Spain; arnau.panisello@iibb.csic.es; 2 Centre Hépato-Biliaire, AP-PH, Hôpital Paul Brousse, 94800 Paris, France; rene.adam@aphp.fr

Ischemia reperfusion injury (IRI) is related to different surgical interventions such as organ resection and transplantation, and therefore its prevention is of great interest. However, several decades of investigations have not, unfortunately, lead to a definitive solution for the treatment of IRI due to its complex and multifactorial pathophysiology with a plethora of underlying mechanisms that are shared among different organs (heart, brain, liver, kidney, etc.) [1,2,3]. The deep exploration of specific IRI pathophysiological and molecular mechanisms is needed to define strategies to reduce its deleterious effects.

IRI is the most important cause of graft failure in transplantation besides immunological rejection. This is the result of cumulative damage that depends on the strength, duration and intensity of the different episodes inherent to transplantation, and leads to the appearance of organ primary/secondary failure, which may happen as a consequence of a re-transplantation [4,5].

Along this Special Issue “Molecular Mechanisms and Pathophysiology of Ischemia-Reperfusion Injury 2.0” we provide a broad overview of the recent insights on several strategies adopted influencing different molecular and pathophysiological mechanisms involved in different organs such as heart, brain, liver and kidney. These strategies in clinical surgery and transplantation embrace the current understanding of some of the underlying pathophysiological molecular pathways and in turn better knowledge of them is determinant to counteract the IRI deleterious effects. Here, we report new advances in IRI molecular mechanisms associated with different strategies, such as ischemic preconditioning (IP) and post-conditioning (Post C) [6,7,8], which are used to reduce the deleterious effects of IRI; including other new approaches based on using micro RNAs (miRNAs) [9,10,11] or mesenchymal cells [12].

It is well-known that the activation of molecular sensors, such as nitric oxide and AMPK, are tied to IP and PostC [13,14,15]. On this line, recent investigations have produced evidence that the action on the melatonin receptor agonists on melatonin 1 (MT1) and 2 (MT2) receptors induced by Ramelteon is an effective heart preconditioning tool against IRI [16]. In addition, recent PostC investigations have shown that AMPK activation is associated with increased glucose metabolism activity in a model of isolated heart after circulatory death [17]. Alternatively, the use of mi-RNAs has been shown to be efficient in countering the deleterious effects of IRI [9,10,11], although it may interfere with the induction of other specific mi-RNAs when pathological conditions such as hypercholesterinemia are present in preconditioned hearts [18].

Moreover, there is growing evidence in the brain that factors such as CELSR1 (Cadherin epidermal growth factor (EGF) laminin G (LAG) seven-pass G-type receptor) are responsible for neuroprotective effects on cerebral ischemia injury [19]. This is consistent with the recent investigations of Schuhmann, M.K. et al. [20] demonstrating that targeting platelet GPVI plus rt-PA administration but not α2β1-Mediated Collagen Binding also limits the deleterious effects of ischemia in mice brain injury.

Recent efforts have been conducted to avoid the detrimental injury effects that occur during liver resection interventions by inhibiting iNOS with L-NAME [21], as well as those associated with the rewarming injury after cold graft preservation, which are relevant in the context of transplantation [22]. With this in mind, several machine perfusion (MP) strategies [23,24,25] have been introduced to better preserve the graft before the subsequent liver transplantation. In general terms, the main goal is to provide vascular flow and oxygen to the donor graft during preservation; either in hypothermic oxygenated perfusion (HOPE) [23,24] (using a perfusion solution, normally Belzer-MPS) or in normothermic MP perfusion (NMP) [25] (using blood or other fluids with oxygen carrying capacity). These strategies can be applied either immediately after organ procurement or after static cold storage (SCS). In any case, the oxygenation of the organs is an unsolved problem that attracts the interest and efforts of researchers in the field [26,27].

Several MP investigations in kidney have shown the benefits of incorporating antioxidants, such as Vectisol, which decreases the early levels of the detrimental radical oxygen species (ROS) [28] or mesenchymal cells, opening a new window to administer cell therapy to damaged organs [29] through MP in different preclinical transplantation models. These promising MP perspectives in kidney [28,29,30] have been extended to liver by using HOPE to rescue vulnerable livers, such as the ones from donors after cardiac death (DCD) and steatotic ones [24,25]. In this sense, the recent investigations by Schlegel et al. [23] have established the protection mechanisms of HOPE based on the metabolism of succinate. However, the establishment of new markers to evaluate the quality of the liver grafts is a pending challenge for the immediate future in IRI investigations [31,32,33]. Recently, a new perfusate named IGL2 containing PEG35 as oncotic agent [34] has been proposed for HOPE. IGL2 benefits are associated with a better preservation of the mitochondria integrity, thus assuring the most suitable restoration of the mitochondrial function and avoiding the inherent redox stress, as suggested by Hofmann et al. [35]. In addition, PEG35 would facilitate the mechano-transduction processes due to its fluid dynamic properties in HOPE [36]. Finally, it is important to consider the potential use of PEG35 as an additive to the perfusates used in NMP, such as blood and solutions containing oxygen carriers. This would be consistent with the benefits of i.v. PEG35 administration in rats subjected to warm ischemia reperfusion [37] where the deleterious effects of IRI were prevented. Further investigations are needed.

In conclusion, the challenge for the upcoming years is to explore in depth the complex molecular pathophysiological mechanisms inherent to IRI in order to establish new frontiers for future interventions in clinical transplantation.
